# Impacts of Time-Fed Concentrate-Based Diets on Plasma Metabolites, Rumen Histology, and mRNA Expression of Hepatic Enzymes of Wethers

**DOI:** 10.3390/ani10040686

**Published:** 2020-04-15

**Authors:** Ghazanfar A. Chishti, Isaac J. Salfer, Krum V. Nedelkov, Tara L. Felix

**Affiliations:** 1 Department of Animal Science, Penn State University, University Park, PA 16802, USA; guc55@psu.edu (G.A.C.); Isaac.Salfer@sdstate.edu (I.J.S.); k_nedelkov@abv.bg (K.V.N.); 2 Dairy and Food Science Department, South Dakota State University, 1224 Medary Ave., Brookings, SD 57006, USA; 3 Faculty of Veterinary Medicine, Trakia University, BG-6000 Stara Zagora, Bulgaria

**Keywords:** acidosis, diet adaptation, nutrition, mRNA expression, sheep

## Abstract

**Simple Summary:**

In modern ruminant meat production systems, wethers and steers are commonly fed diets containing 80% grain, or more. These diets are commonly fed to increase fat deposition in meat-producing ruminants. However, when wethers and steers are not appropriately transitioned to grain-based diets, they can experience metabolic and inflammatory conditions that negatively affect health and production. It is still unclear how meat-producing ruminants adapt to grain-based diets over time. This study evaluated the effects of an abrupt dietary change, from 80% forage to 80% grain, on rumen, plasma, and liver metabolism in growing wethers and monitored their ability to adapt over time. The results of the study suggest that wethers fed an 80% grain diet adapt over time by altering the expression of key enzymes involved in the systemic inflammation, iron metabolism, and cholesterol and glucose synthesis. This study provides novel insight into the physiology of fattening ruminants that have been abruptly fed grain-based diets and highlights the fact that meat-producing animals can be fed grain-based diets to meet increasing human meat requirements.

**Abstract:**

Transition to grain increases inflammation and causes parakeratosis, which can decrease growth performance in fattening animals. It is unknown if ruminants adapt to these inflammatory responses over time. In a three-phase, 49-day experiment, all wethers (*n* = 13, BW = 50.6 ± 4.7 kg; 4.9 ± 0.3 months of age) were fed an 80% forage diet during P1(day 0 to 21). On day 21, 4 wethers were slaughtered to obtain baseline liver and rumen tissue. During P2 (day 22 to 25), the remaining wethers were fed an 80% concentrate diet. Four wethers were slaughtered on day 25 to obtain P2 liver and rumen tissue. During P3 (day 22 to 49), the remaining five wethers were fed 80% concentrate diets and were slaughtered on day 49 to obtain P3 liver and rumen tissue. Rumen parakeratosis was greater (*p* ≤ 0.02) in wethers sampled in P2 and P3 when compared to those sampled in P1. Among positive acute phase reactants, expression of serum α-amyloid (*SAA*) and haptoglobin (*HPT*) tended (*p* ≤ 0.12) to be 6- and 10-fold greater, respectively, in wethers sampled in P2 compared to wethers sampled in P1; however, *SAA* and *HPT* expression was not different between wethers sampled in P3 and P1. Plasma glucose and β-hydroxybutyric acid (BHBA) increased (*p* ≤ 0.03) in wethers sampled in both P2 and P3 compared to the wethers sampled in P1, while total protein and cholesterol decreased (*p* ≤ 0.06) only in wethers sampled from P2 compared to those sampled in P1. Hepatic acute phase responses suggest that the wethers adapted to an 80% concentrate diet over time.

## 1. Introduction

In modern ruminant production systems, wethers and steers are commonly transitioned to diets containing 80% grain, or more, during the finishing phase. In situations where these animals are not appropriately transitioned to concentrate-based diets, ruminal acidosis can occur [1]. Ruminal acidosis can negatively affect the integrity and barrier function of the rumen epithelium by increasing parakeratosis, vacuolation, and cell degeneration [2,3] The loss of barrier function in the rumen epithelium can lead to the translocation of bacteria, and its endotoxins (lipopolysaccharides), from the rumen into the systemic circulation and result in a hepatic acute phase response [4]. The acute phase response is caused by systemic inflammation and is characterized by the increase or decrease in acute phase reactants. Among acute phase reactants, haptoglobin, serum α-amyloid, and hepcidin increase, while albumin and cholesterol decrease during acute phase response [5,6]. During the acute phase response, hepatic gluconeogenesis increases to meet the elevated glucose needs of the immune system, and hepatic ketogenesis decreases to support gluconeogenesis [7]. In goats, acidosis elicited an acute phase response when only 60% concentrate was fed [8]. There is a lack of information regarding the impact of inflammatory responses to acidosis in fattening ruminants abruptly fed grain-based diets. 

Therefore, the objectives of this study were to determine the impacts of an abrupt dietary change from an 80% forage to an 80% concentrate-based diet on rumen histology, plasma metabolites, and mRNA expression of key hepatic enzymes and proteins in growing wethers. It was hypothesized that after 4 days of feeding a grain-based diet, the acute phase response would be increased, but that wethers could adapt to the grain-based diet after 28 days.

## 2. Materials and Methods 

All procedures for animal use were approved by the Pennsylvania State University Institutional Animal Care and Use Committee (#47773). This experiment was conducted at the Pennsylvania State University Beef and Sheep Center (State College, PA, USA). 

### 2.1. Animal and Diet Management

Two diets were fed in this experiment, 80% forage and 80% concentrate on a dry matter (DM) basis. Diets were formulated using grass hay, dry rolled corn, soybean meal, urea, and vitamin/mineral supplements (Table 1) to meet the requirements of the wethers fed in this study [9]. Samples of dietary ingredients were collected on days 0, 25, and 49 of the experiment for subsequent analysis. The individual ingredients were dried for 48 h at 55 °C, then ground through a Wiley mill (1 mm screen, Arthur H. Thomas, Philadelphia, PA, USA) Ground samples were analyzed for neutral detergent fiber (NDF) (using Ankom Technology method 6; Ankom200 Fiber Analyzer, Ankom Technology, Macedon, NY, USA), crude protein (CP) (using a Costech ECS 4010 C/N/S elemental analyzer; Costech Analytical Technologies Inc., Valencia, CA, USA), and starch [10].

The 49-day experiment used 13 crossbred Hampshire × Suffolk wethers (Body weight (BW)= 50.6 ± 4.7 kg; 4.9 ± 0.3 months of age). The 13 wethers were assigned to slaughter groups at the start of the study, such that all wethers within a slaughter group had the same BW at the initiation of the study. These slaughter groups included wethers to be slaughtered for baseline sampling (on day 21), abrupt response to acidosis challenge (on day 25), and chronic response to acidosis challenge (on day 49). Wethers were then fed in 3 phases: P1 (day 0 to 21), P2 (day 22 to 25), and P3 (day 22 to 49). During P1, all wethers (*n* = 13) were fed the 80% forage diet. On day 21, 4 wethers were slaughtered 4 h after morning feeding to obtain samples of liver and rumen tissue that would be used to establish a baseline for wethers sampled in P2 and P3. On day 22, the remaining 9 wethers were fed an 80% concentrate diet. On the last day of the P2 phase (day 25), 4 wethers were slaughtered 4 h after morning feeding to obtain P2 samples of liver and rumen tissue to determine the abrupt changes caused by feeding a concentrate-based diet. The remaining 5 wethers were fed 80% concentrate-based diets until day 49 when those wethers were slaughtered 4 h after morning feeding to obtain P3 samples of liver and rumen tissue used to determine if wethers could adapt to dietary changes given time. Due to the need to slaughter animals to obtain samples over time, there was a lack of a contemporary group fed 80% forage-based diet for the duration of this study. Using P1 wethers as a baseline allowed the number of animals needing to be sacrificed to be minimized. In addition, wethers fed in this study were old enough to be considered mature and were all housed in an environmentally controlled barn. Therefore, these conditions allowed the changes observed to represent dietary factors, rather than other environmental or developmental factors.

During P1, all wethers were fed for ad libitum intakes. In the P2 phase, wethers were offered the same amount of feed on a DM basis that was offered to the wethers slaughtered in P1 phase. After the P2 phase, wethers were again fed for ad libitum intakes for the duration of the study. Throughout the experiment, wethers were fed equal portions of the ration twice daily at 08:30 and 16:30 h and were housed in a mechanically ventilated and temperature-controlled barn. Wethers were housed in groups during P1 due to the lack of dietary differences. One week prior to the dietary change from the 80% forage to the 80% concentrate diet, the wethers stratified for slaughter in P2 were housed and fed individually. The P2 wethers continued to be fed individually until slaughter for a total of 10 days individual feeding. The wethers in P3 were also fed and housed individually 1 week prior to slaughter. The greatest individual intake variation was expected in wethers fed 80% concentrate diet; therefore, wethers in P2 and P3 were fed individually at least 1 week prior to slaughter.

### 2.2. Sample Collection and mRNA Expression

Wethers were stunned with a captive bolt and exsanguinated on day 21, 25, and 49 to obtain samples for P1, P2, and P3, respectively. Liver and rumen tissue were obtained within 15 min of exsanguination. Liver tissue was collected from 2 to 3 sites on the right lobe, rinsed with cold water, flash-frozen in liquid nitrogen, and stored at −80 °C. Liver tissue was analyzed for mRNA expression of pyruvate carboxylase (*PC*); 3-hydroxy-3-methyl-glutaryl CoA-synthase (*HMGCS-2*); acute phase reactant proteins haptoglobin (*HPT*), serum α-amyloid (*SAA*), hepcidin (*HAMP*), albumin (*ALB*); and 3-hydroxy-3-methylglutaryl-CoA reductase (*HMGCR*) through real-time quantitative reverse transcriptase PCR.

The expression of 2 candidate reference genes, *ACTB* and *PPIA*, previously used for normalization of liver gene expression, were also examined [11,12]. Approximately 60 mg of tissue was homogenized using an electric homogenizer (Polytron PT 10-35; Kinematica GmbH, Lucerne, Switzerland) in 1.5 mL of RNA-Solv (Omega Bio-Tek, Inc., Norcross, GA, USA). Total RNA was isolated using phenol/chloroform extraction buffer and purified on a silica column (E.Z.N.A. Total RNA Mini Kit II, Omega Bio-tek, Inc.) with on-column DNase treatment (DNASE70-1SET, Sigma–Aldrich, St. Louis, MO, USA) according to the procedure of Chishti et al. [13]. Purified RNA was quantified at 260 nm using a spectrophotometer (NanoDrop 2000, Thermo Fisher Scientific, Waltham, MA, USA). The RNA purity was assessed by absorbance ratio of 260/280 (2.07 ± 0.02; mean ± SD), while its integrity (RQI: 9.43 ± 0.51; mean ± SD) was measured with an Experion Bioanalyzer (Bio-Rad Laboratories Inc., Hercules, CA, USA). A total of 1 μg of RNA from each sample was reverse transcribed to cDNA (High Capacity Reverse Transcriptase kit, Applied Biosystems, Foster City, CA, USA). The abundance of genes of interest was quantified in triplicate using real-time PCR (7500 Fast Real-time PCR system, Applied Biosystems, Foster City, CA, USA) with 400 nM of forward and reverse primers using SYBR Green reagent as a reporter (PerfeCTa SuperMix with ROX, Quanta Biosciences, Gaithersburg, MD, USA). Forward and reverse primers were designed to amplify a product length between 70 and 200 bp using Primer-BLAST (NCBI; Table 2).

All experimental samples for each gene were run on the same plate. The conditions for PCR reactions were as follows: enzyme activation, 10 min at 95 °C; cycling stages with 40 cycles, at 95 °C for 15 s and 60 s at 60 °C. Melt curve analysis was conducted after each run to verify the presence of a single qPCR product. Efficiencies of PCR for each primer pair and R^2^ for each standard curve were ≥98% and >0.98, respectively (Table 2). The expression of reference genes was ranked by stability values using major computational programs (geNorm, BestKeeper, and the comparative Delta-Ct method) through RefFinder software [14] (Table 3). The combination of *ACTB* and *PPIA* were found to have similar stability. 

In addition to the liver samples, rumen samples were also collected at slaughter. Ruminal contents were removed from the rumen, and approximately 5 cm^2^ was obtained from the ventral ruminal sac and preserved in 4% paraformaldehyde solution for subsequent histomorphology processing. Rumen tissue samples were dehydrated and cleared by a series of graded ethanol (70, 85, 95, and 100%), and xylene substitute (Histosolve, Thermo Fisher Scientific) washes and embedded in paraffin in an automatic tissue processor (Leica TP1020, Leica Biosystems Inc. Buffalo Grove, IL, USA). Perpendicular sections were stained with hematoxylin and eosin in autostainer (Leica Autostainer XL, Leica Biosystems Inc. Buffalo Grove, IL, USA) for observation under the light microscope. For calculating surface area, length and width of all the rumen papillae from a slide were determined. To determine papillae health, the width of both the dense and translucent corneum keratin layers was measured as defined by Steele et al. [15]. In brief, the dense keratin layer was composed of keratinized squamous cells of the corneum layer, while the translucent keratin layer contained vacuolated and translucent cells of the corneum layer. These measurements were taken using an eyepiece camera mounted on the light microscope OMAX (A3550S) equipped with computer-aided image analysis software (ToupView 3.7, Hangzhou ToupTek Photonics Co., Zhejiang, China). Papillae surface area was calculated as papillae height × papillae width [16].

Blood (10 mL) was collected via the jugular vein into heparin-coated evacuated tubes (BD Ref # 366480, Franklin Lakes, NJ, USA) 4 h after morning feeding on day 20, 24, and 48, the final day before slaughter within each phase. To determine hematocrit, approximately 0.5 mL of blood was centrifuged in capillary tubes and then read under a microhematocrit reader (Dickinson and Company, Parsippany, NJ, USA) [17] The remainder of the blood was centrifuged at 3000× *g* for 15 min at 4 °C. After centrifugation, plasma was pipetted into microtubes and frozen (−20 °C) for analysis of plasma β-hydroxybutyric acid (BHBA), total protein, glucose, and total cholesterol. Plasma BHBA was determined on samples using the Stanbio BHBA Liqui-Color kit (Procedure No. 2440, Stanbio Laboratory, Boerne, TX, USA). Plasma total protein was determined using a drop of plasma under a refractrometer (ATAGO CO., LTD, Tokyo, Japan) [18]. Plasma glucose and cholesterol were determined using Stanbio kits procedure no. 1070 and 1010, respectively (Stanbio Laboratory, Boerne, TX, USA).

### 2.3. Statistical Analysis

Results of rumen histology, plasma parameters, and mRNA expression of genes were analyzed by PROC MIXED in SAS (SAS v. 9.4 Inst., Cary, NC, USA). Animal was used as the experimental unit. The experimental phase was used as a fixed effect to determine the impact of feeding concentrate-based diets over time. Expression data of each gene was normalized by the Box–Cox procedure in Minitab (Table S1). Least-squares means and standard error of means for *HPT* and *SAA* expression were back-transformed for the presentation of results (Table S2). Least-square means were separated using preplanned contrast of wethers sampled in P1 versus P2 and P1 versus P3. Significant differences were declared at *p* ≤ 0.05, while tendencies were discussed between 0.05 ≤ *p* ≤ 0.15 [19,20]. To analyze the variance in data, mRNA expression of all the enzymes was analyzed for principal component analysis (PCA) using JMP software (SAS Inst. Inc., Cary, NC, USA). Principal component analysis confirmed low variability among the samples, with component 1 and component 2 explaining 59% of the variability.

## 3. Results and Discussion

In lactating ruminants, diets containing 60% or more concentrate (on a DM basis) can cause ruminal acidosis and initiate a hepatic acute phase response [4,8]. However, there is no definitive information available regarding what inclusion of concentrate in wether diets can cause these inflammatory responses. In the current experiment, wethers were abruptly challenged with an 80% concentrate-based diet to examine the effects on rumen histology, plasma metabolites, and mRNA expression of key hepatic enzymes and proteins.

Histopathology of rumen epithelium is a reliable method for studying and confirming ruminal acidosis [21]. Ruminal acidosis negatively affects rumen epithelium morphology, increasing parakeratosis, vacuolation, and cell degeneration [2]. Parakeratosis of rumen epithelium has long been characterized by the accumulation of dense and compact keratinized cells in the corneum layer [22]. This description of parakeratosis has been generally applied to both cattle and sheep [23]. However, Steele et al. [15] proposed defining a new term, “translucent hyperkeratosis”, when they reported loosely connected translucent and vacuolated cells in lambs fed grain-based diets. In the present study, the width of both the dense squamous corneum layer, traditional of ruminal parakeratosis, and the translucent keratin layer, according to the updated definition proposed by Steele et al. [15], were measured in all three phases. While no differences (*p* ≥ 0.16) in the width of dense squamous corneum layer were observed in any of the phases, the width of translucent keratin layer increased (*p* ≤ 0.02) by 142% and 336% in wethers sampled in P2 and P3, respectively, compared to the wethers sampled in P1 (Table 4). These results are similar to the 202% increase in the width of translucent keratin layer in lambs fed 79% concentrate diet compared to the lambs fed 29% concentrate diet reported by Steele et al. [15], confirming the conclusion of the previous authors that ruminal parakeratosis in lambs fed grain-based diets may not fit the traditional definition of a dense squamous corneum layer.

In the current study, rumen papillae were of similar (*p* ≥ 0.37) length regardless of the sampling phase, while they were 47% wider (*p* = 0.02) in wethers sampled in P3 compared to the wethers sampled in P1. The greater papillae width increased rumen papillae surface area (*p* = 0.04) by 71% in wethers sampled in the P3 compared to the wethers sampled in P1 (Table 4). The increased width of translucent keratin layer might have contributed to the increased papillae width and surface area in wethers sampled in P3 compared to the wethers sampled in P1. The increase in papillae surface area in the wethers sampled in P3 was likely due to an increase in short-chain fatty acids (SCFA) production. Increasing concentrates fed in the diet consistently increase total SCFA production [24]. The increase in SCFA, especially butyrate, has been associated with increases in papillae growth [25,26]. Although, there was a tendency (*p* = 0.09) for 34% greater papillae width in wethers sampled in P2 compared to wethers sampled in P1, no difference (*p* = 0.36) was found in papillae surface area of wethers sampled in P2 compared to wethers sampled in P1, suggesting there may not have been enough time to stimulate rumen papillae growth in wethers sampled in P2. 

**Figure 1:**
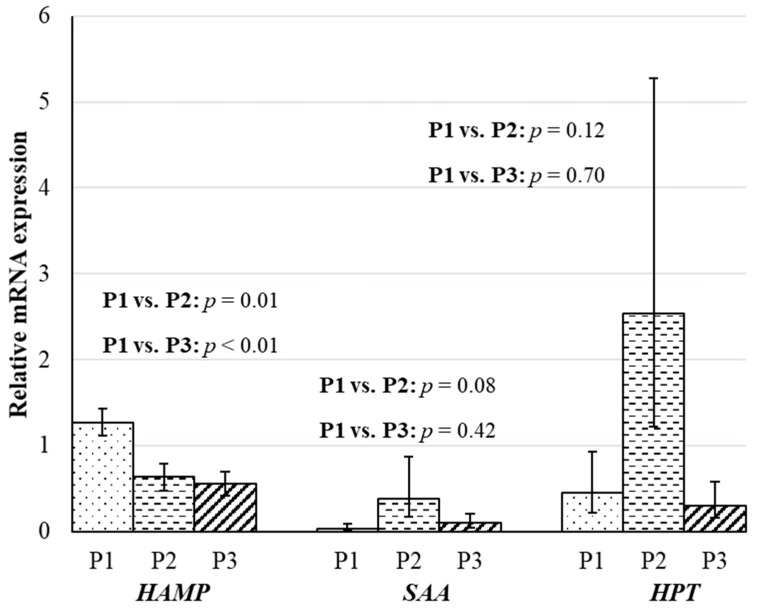
Impacts of time-fed concentrate-based diets on relative mRNA expression of hepatic positive acute phase reactants of wethers. The experiment was divided into three phases: P1 (day 0 to 21), P2 (day 22 to 25), and P3 (day 22 to 49). During P1, all wethers (*n* = 13) were fed the 80% forage diet. On day 22, the remaining nine wethers were fed an 80% concentrate diet. The contrasts between wethers sampled in P1 (*n* = 4) and P2 (*n* = 4) and between wethers sampled in P1 (*n* = 4) and P3 (*n* = 5) on least square means of relative mRNA expression of Hepcidin (*HAMP*), Serum α-amyloid (*SAA*) and Haptoglobin (*HPT*) in liver tissue. Least-squares means and standard error of means for *HPT* and *SAA* expression were back-transformed for the presentation of results. Least-squares means and standard error of means before back transformation for *SAA* and *HPT* are presented in S2.

Greater concentrate-based feeding can cause parakeratosis and vacuolation, thereby decreasing the barrier function of rumen epithelium and initiating hepatic acute phase response [3,4]. In the current study, there was a tendency for a 6-fold greater relative hepatic mRNA expression of *HPT* (*p* = 0.12) and 10-fold greater relative hepatic mRNA expression of *SAA* (*p* = 0.08), when wethers sampled in P2 were compared to the wethers sampled in P1 (Figure 1). Minuti et al. [27] observed an 8-fold increase in plasma concentrations of HPT in rams after 3 days of an acute acidosis challenge over baseline samples. A wide range of individual responses to grain challenges has been reported [28], which may be the reason why there was only a tendency for greater *HPT* and *SAA* mRNA expression in wethers sampled in P2 when compared to wethers sampled in P1. For example, in lactating goats, increased mRNA expression and plasma concentration of *HPT* and *SAA* have been observed even after 6 to 9 weeks of consuming a 60% concentrate-based diet [8,29]. Contrary to the results in lactating goats, wethers sampled in P1 and P3 had similar (*p* ≥ 0.42) mRNA expression or both *HPT* and *SAA* suggesting that wethers in the current study physiologically adapted to 80% concentrate-based diet after 28 days (Figure 1).

Unlike *HPT* and *SAA*, mRNA expression of *HAMP*, decreased (*p* < 0.05) by 2.0- and 2.3-fold in wethers sampled in P2 and P3, respectively, when compared to wethers sampled in P1 (Figure 1). Similar to the downregulation of *HAMP* expression, hematocrit concentrations decreased (*p* < 0.01) by 8% and 7% in wethers sampled from P2 and P3, respectively, when compared to the wethers sampled during the P1 (Table 5). Hepcidin, or *HAMP*, is the key hormonal regulator for iron homeostasis, and plasma levels of iron and mRNA expression of *HAMP* generally react in an inverse manner [30,31]. Because of the close relationship, the synthesis of *HAMP* appears to be regulated by plasma iron and the production of erythrocytes [31]. It is likely then that the anemia, indicated by the decreased hematocrit, may have caused the downregulation of *HAMP* mRNA expression as a compensatory feedback mechanism to increase iron availability for erythropoiesis [32,33]. The decrease in *HAMP* mRNA expression can be an important hepatic adaptation for countering anemia in wethers challenged with concentrate-based diets. 

**Figure 2:**
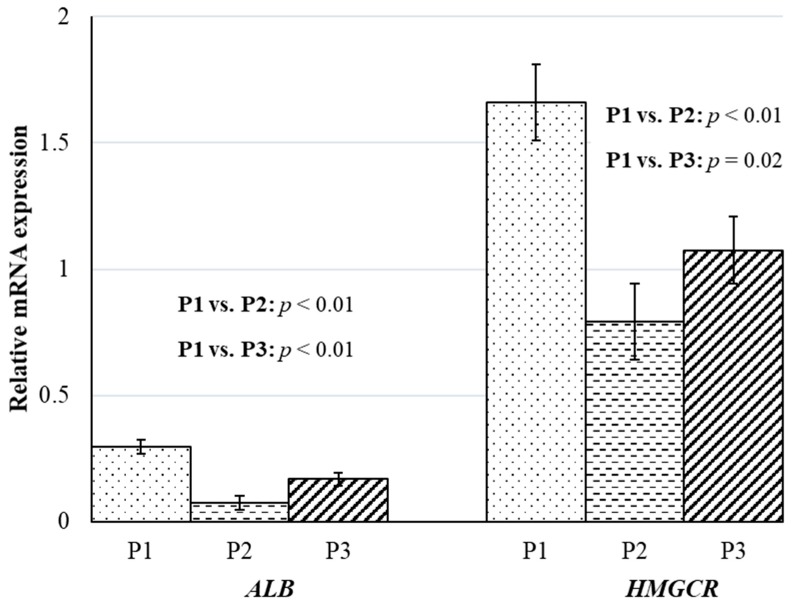
Impacts of time-fed concentrate-based diets on relative mRNA expression of hepatic negative acute phase reactants of wethers. The experiment was divided into three phases: P1 (day 0 to 21), P2 (day 22 to 25), and P3 (day 22 to 49). During P1, all wethers (*n* = 13) were fed the 80% forage diet. On day 22, the remaining nine wethers were fed an 80% concentrate diet. The contrasts between wethers sampled in P1 (*n* = 4) and P2 (*n* = 4) and between wethers sampled in P1 (*n* = 4) and P3 (*n* = 5) on LS means of relative mRNA expression of Albumin (*ALB*) and 3-hydroxy-3-methylglutaryl-CoA reductase (*HMGCR*).

Hepatic mRNA expression of *ALB* also decreased (*p* < 0.05), similar to *HAMP* (Figure 2). There was a 4.0-fold decrease in *ALB* mRNA expression in wethers sampled in P2 and 1.8-fold in wethers sampled in P3 when compared to the wethers sampled in P1. Albumin contributes more than 50% of total plasma protein, and changes in concentrations of *ALB* are often reflected in total plasma protein. However, in the current study, total plasma protein concentration decreased (*p* < 0.01) by 6% in wethers sampled in P2 when compared to wethers sampled in P1; but was not different (*p* = 0.47 between wethers sampled in P1 and P3. Similar to the current study, an 11% decrease in plasma *ALB* concentration was observed in rams after a 72 h, or acute, acidosis challenge [27]. But, unlike the current study, cows continued to experience an 11% decline in total plasma protein concentration even after 8 weeks of feeding a 60% concentrate diet [4]. In addition, even though *ALB* expression was still reduced in wethers sampled from P3, when compared to those sampled in P1, the reduction was not as severe as the reduction when P2 samples were compared to P1. The decrease in the severity of the *ALB* response and lack of change between P1 and P3 total protein concentrations also suggests that wethers may have been overcoming the acidotic challenge or adapting physiologically.

Cholesterol is another important acute phase reactant [34]. In the current study, plasma cholesterol concentration tended to decrease by 18% (*p* = 0.06) in wethers sampled in the P2 compared to the wethers sampled in P1, indicating an increased acute phase response. On the contrary, we did not observe any decrease in plasma cholesterol concentration in wethers sampled in P3; rather, there was a 48% increase (*p* < 0.01) in plasma cholesterol concentration in wethers sampled in P3 compared to the wethers sampled in P1 (Table 5). Minuti et al. [27] reported a 47% decrease in plasma cholesterol concentration in rams after 72 h of acidosis challenge. These authors did not report a longer term effect on cholesterol. While the greater plasma cholesterol in wethers sampled in P3 in the current study could be due to the increased availability of ruminal short-chain fatty acids (SCFA) for cholesterol synthesis, several studies in the literature have associated increased production of SCFA with the feeding of concentrate-based diets [24]. It could also be an adaptation, as noted with several other acute phase reactants in the current study. Decreased expression of cholestrogenic genes in rumen epithelium is considered a cellular adaptation to ruminal acidosis [35] because intracellular cholesterol is homeostatically regulated to reduce cell damage caused by this acute phase reactant. In the current study, mRNA expression of *HMGCR,* a rate-limiting cholestrogenic enzyme, was downregulated (*p* < 0.05) by 2.1- and 1.5-fold in both the P2 and P3 wethers, respectively, when compared to wethers sampled in P1 (Figure 2). The reduction in circulating cholesterol in P2 samples and the downregulation of *HMGCR* expression in the liver suggest that cholesterol synthesis was being regulated during an acute response to ruminal acidosis and that the regulation of cholesterol synthesis began in the liver.

**Figure 3:**
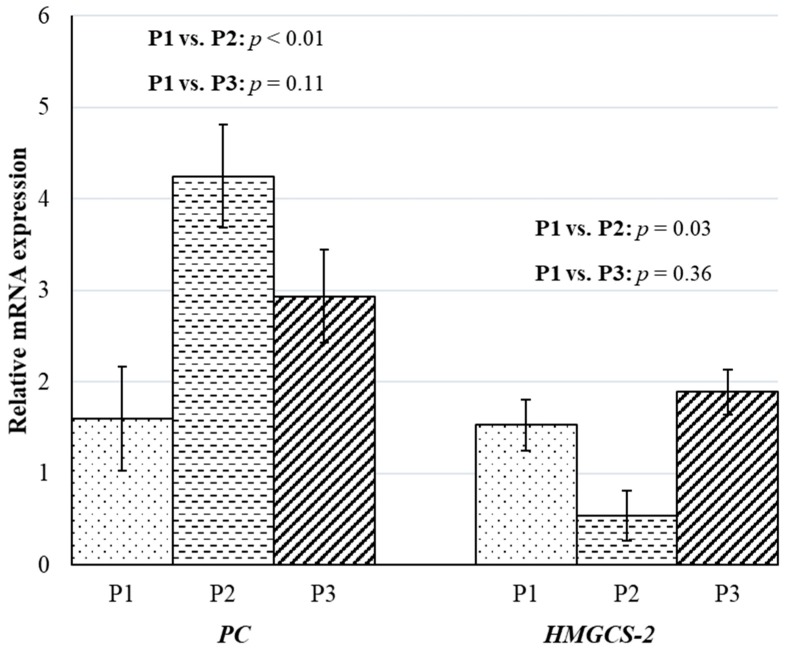
Impacts of time-fed concentrate-based diets on relative mRNA expression of rate-limiting gluconeogenic and ketogenic enzymes of wethers. The experiment was divided into three phases: P1 (day 0 to 21), P2 (day 22 to 25), and P3 (day 22 to 49). During P1, all wethers (*n* = 13) were fed the 80% forage diet. On day 22, the remaining nine wethers were fed an 80% concentrate diet. The contrasts between wethers sampled in P1 (*n* = 4) and P2 (*n* = 4) and between wethers sampled in P1 (*n* = 4) and P3 (*n* = 5) on LS means of relative mRNA expression of Pyruvate carboxylase (*PC*) and 3-hydroxy-3-methyl-glutaryl CoA-synthase (*HMGCS-2*) in liver tissue.

Previous experiments have demonstrated that plasma glucose concentrations increase by as much as 6% during ruminal acidosis caused by increased concentrate feeding [36]. In the current study, glucose concentrations were 39% and 41% greater (*p* < 0.01) in wethers sampled from P2 and P3, respectively, when compared to the wethers sampled during the P1 (Table 5). Concomitant with the increase in plasma glucose, mRNA expression of pyruvate carboxylase, or *PC*, the key rate-limiting enzyme for lactate gluconeogenesis, was increased. The mRNA expression of *PC* and lactate turnover to glucose are strongly correlated [37]. In the current study, the expression of *PC* was 2.6-fold greater (*p* < 0.01) in wethers sampled in P2 compared to the wethers sampled in P1; and, there was a tendency (*p* ≤ 0.11) for a 1.8-fold greater expression of *PC* in wethers sampled in P3 compared to the wethers sampled in P1 (Figure 3). The liver can adapt to the use of amino acids and lactate for gluconeogenesis during situations of negative energy balance or when the rumen is underdeveloped [38]. The mRNA expression of *PC* likely increased in the wethers sampled in the P2 and P3 phases as compared to the wethers sampled in the P1 phase as an adaptation to utilize lactate produced from rumen [39].

The acute phase response, in the current study likely also led to the 2.8-fold reduction (*p* = 0.03) in mRNA expression of *HMGCS-2*, the rate-limiting ketogenic enzyme, in wethers sampled in P2 compared to wethers sampled in P1 (Figure 3) as a means to conserve additional glucose [7]. However, there was no difference (*p* = 0.36) in the expression of *HMGCS-2* between wethers sampled in P3 and the wethers sampled in P1. Even though *HMGCS-2* responses would indicate wethers were not suffering from ketosis, the plasma BHBA concentration was 45% and 113% greater (*p* < 0.01) in wethers sampled in P2 and P3, respectively, when compared to the wethers sampled during P1. In ruminants, BHBA is produced from both the rumen epithelium and the liver. In the fed state, the rumen epithelium is the principal source of BHBA, while during fasting and negative energy, balanced hepatic ketogenesis, or production of BHBA in the liver, predominates. In the current experiment, intake of wethers fed in P2 (dry matter intake = 2.38 ± 0.21% of BW) and P3 (dry matter intake = 2.22 ± 0.18% of BW) was more than their maintenance requirements and wethers never experienced negative energy balance, so the increase in plasma BHBA concentration in wethers sampled in both P2 and P3 was most likely from the rumen epithelium. This greater BHBA production from rumen epithelium may be associated with ruminal acidosis resistance [40]. Similar to our study, plasma BHBA concentrations were 160% greater in lambs fed a 79% concentrate diet when compared to the lambs fed a 29% concentrate diet [15]. However, in lactating cows, decreased plasma BHBA concentration has been related to increased rumen endotoxin concentrations due to increased concentrate feeding [34]. The discrepancy between the experiments may be attributed to the difference in the stage of production and species between the two experiments. 

This study has some limitations that may call in to question the results. One of the first challenges was that the study lacked a traditional, contemporary control group. While this may suggest age could play a role in the results, all wethers in the study were mature, and there were only 28 days between the time P1 wethers were slaughtered compared to when P3 wethers were slaughtered. Therefore, we expect the effects of maturity to be minimal. Additionally, this study reported mRNA expression rather than changes in protein activity or function. The authors recognize that other molecular changes, including translational efficiency and post-translational modifications, can change the relationship between mRNA concentrations and function. However, for the enzymes measured, several previous studies have reported strong relationships between mRNA expression and functional activity [37,41]. Future experiments investigating the effects of abrupt acidosis challenge on inflammation should include a contemporary control group and consider measuring changes in protein expression and protein phosphorylation.

## 4. Conclusions

The results of the current study suggest short-term feeding of a concentrate-based diet increased liver inflammatory response, but these responses were mitigated after an additional 27 d feeding of concentrate-based diets. These data together suggest that wethers may adapt to the 80% concentrate-based diet over time by altering the expression of key enzymes involved in the acute phase response, iron metabolism, cholesterogenesis, and gluconeogenesis. However, translucent keratin was elevated during both short-term and long-term 80% concentrate-based feeding, suggesting that rumen epithelium damage remained despite changes in other physiologic responses. These results provide novel insights into liver and rumen epithelial function in ruminants abruptly fed grain-based diets. 

## Figures and Tables

**Table 1:** Composition of diets fed to wethers.

Item, % DM Basis	Forage	Concentrate
Ingredients		
Grass hay ^1^	80.00	20.00
Dry rolled corn	8.10	69.80
Soybean meal	10.15	8.45
Mineral and Vitamin Supplement ^2^	0.75	0.75
Urea	0.50	0.50
NH_4_Cl	0.50	0.50
Analyzed nutrient composition		
DM	85.7	85.7
NDF	56.8	20.7
ADF	29.9	9.3
Starch	7.1	47.8
CP	14.1	11.9

^1^ Grass hay composition: 69.0% neutral detergent fiber (NDF), 36.6% acid detergent fiber (ADF), and 8.57% crude protein (CP). ^2^ Mineral and Vitamin premix composition (min values): Ca 16.54%, P 4.00%, NaCl 14.40%, Cl 10.07%, Na 5.06%, Mg 2.00%, K 0.500%, S 1.00%, Co 20.00 mg/kg, Cu 0.00 mg/kg, I 80.00 mg/kg, Fe 1797.41 mg/kg, Mn 1500 mg/kg, Se 38 mg/kg, Zn 1500 mg/kg, Niacin 1.81 mg/kg, Choline 35.50 mg/kg, Vit A 400,800 IU/kg, Vit D 58,750 IU/kg, Vit E 227 IU/kg.

**Table 2:** Gene, primer sequence, access code, and base pair length according to NCBI, along with QPCR correlation and efficiency.

Gene	Primer	Access Code	Base Pair Length	R^2^	Efficiency
*ACTB*	F’-CATCGGCAATGAGCGGTTCC R’-CCGTGTTGGCGTAGAGGT	NM_001009784	146	1	108.1
*PPIA*	F’-TGTGCCAGGGTGGTGACTTCA R’-TGCTTGCCATCCAACCACTCAG	AY251270	196	0.999	100.3
*SAA*	F’-TGATCAGTGATGCCAGAGAAAAT R’-TCTGAAGTGGTTGGGGTCTTT	XM_004019477.3	146	0.997	100.3
*HPT*	F’-TGAGGCAGTGTGCGGAAAG R’-ATCCAGCGACCCACCTATGA	XM_012110686.2	90	0.998	102.3
*ALB*	F’-TAGCTCGCCTGAGCCAGAAA R’-GCAAGATCTGCCCTGTCGTC	NM_001009376.1	136	0.996	98.0
*PC*	F’-GAGGAGATTACCGATGTGGACC R’-GGAACACCTCGATGCGGC	XM_027959895.1	184	0.999	106.0
*HMGCS*	F’-GTCCTCGAGAGAGGGCTTAGA R’-ATTTGTCCAGGGCCCGTAAG	XM_004002390.4	133	0.986	100.6
*HMGCR*	F’-TGCAGATGGGATGACTCGTG R’-TGTAGACGTGCAAACCTGCT	XM_004010192.4	144	0.999	105.3
*HAMP*	F’-AGACACGACAGCTCACAGAC R’-ATGGGAAAGTGGGTGTCTCG	NM_001195312.1	103	0.995	105.3

**Table 3:** Stability values of candidate reference genes according to three different evaluation methods used by RefFinder.

Stability Values ^1^	*ACTB*	*PPIA*
Delta CT method	0.36	0.36
BestKeeper	0.59	0.66
Genorm	0.36 *	
Comprehensive ranking value	1.19	1.41

^1^ Least value gives the highest ranking to candidate genes. * indicates combine stability value of *ACTB* [12] and *PPIA* [11].

**Table 4:** Impacts of time-fed concentrate-based diets on rumen histology of wethers.

Parameter	Phases ^1^			SEM ^2^	*p*-Value	
	P1	P2	P3		P1 vs. P2	P1 vs. P3
n, wethers	4	4	5	-	-	-
Dense keratin ^3^, μm	19.7	21.8	32.6	6.32	0.95	0.16
Translucent keratin ^4^, μm	22.6	54.7	98.7	8.48	0.02	0.01
Papillae width, mm	0.350	0.465	0.512	0.044	0.09	0.02
Papillae length, mm	1.16	1.15	1.36	0.165	0.98	0.37
Papillae area, mm^2^	0.410	0.530	0.696	0.0900	0.36	0.04

^1^ The experiment was divided into three phases: P1 (day 0 to 21), P2 (day 22 to 25), and P3 (day 22 to 49). During P1, all wethers (*n* = 13) were fed the 80% forage diet. On day 22, the remaining nine wethers were fed an 80% concentrate diet. ^2^ SEM = Standard error of means ^3^ Dense keratin layer was composed of keratinized squamous cells of the corneum layer. ^4^ Translucent keratin layer contained vacuolated and translucent cells of the corneum layer.

**Table 5:** Impacts of time-fed concentrate-based diets on plasma metabolites of wethers.

Parameter	Phase ^1^			SEM ^2^	*p*-Value	
	P1	P2	P3		P1 vs. P2	P1 vs. P3
n, wethers	4	4	5	-	-	-
BHBA ^3^, mg/dL	2.88	4.16	6.18	0.543	0.03	<0.01
Glucose, mg/dL	62.3	86.8	87.9	2.98	<0.01	<0.01
Hematocrit, %	36.8	33.7	34.2	1.02	<0.01	<0.01
Cholesterol, mg/dL	50.0	41.2	73.6	4.77	0.06	<0.01
Total Protein, g/dL	6.23	5.76	6.14	0.127	<0.01	0.47

^1^ The experiment was divided into three phases: P1 (day 0 to 21), P2 (day 22 to 25), and P3 (day 22 to 49). During P1, all wethers (*n* = 13) were fed the 80% forage diet. On day 22, the remaining nine wethers were fed an 80% concentrate diet. ^2^ SEM = Standard error of means. ^3^ BHBA represents ketone body β-hydroxybutyric acid.

## Supplementary Materials

The following are available online at https://www.mdpi.com/2076-2615/10/4/686/s1, Table S1: Box-Cox transformation received by mRNA expression of genes used in this study, Table S2: Least square means ± SEM of mRNA expression of Serum α-amyloid and Haptoglobin before back transformation.

## References

[1] Owens F.N., Secrist D.S., Hill W.J., Gill D.R. (1998). Acidosis in cattle: A review. J. Anim Sci..

[2] Ahrens F.A. (1967). Histamine, lactic acid, and hypertonicity as factors in the development of rumenitis in cattle. Am. J. Vet. Res..

[3] Steele M.A., AlZahal O., Hook S.E., Croom J., McBride B.W. (2009). Ruminal acidosis and the rapid onset of ruminal parakeratosis in a mature dairy cow: A case report. Acta Vet. Scand..

[4] Guo J., Chang G., Zhang K., Xu L., Jin D., Bilal M.S., Shen X. (2017). Rumen-derived lipopolysaccharide provoked inflammatory injury in the liver of dairy cows fed a high-concentrate diet. Oncotarget.

[5] Baumann H., Gauldie J. (1994). The acute phase response. Immunol. Today.

[6] Murata H., Shimada N., Yoshioka M. (2004). Current research on acute phase proteins in veterinary diagnosis: An overview. Vet. J..

[7] Memon R.A., Feingold K.R., Moser A.H., Doerrler W., Adi S., Dinarello C.A., Grunfeld C. (1992). Differential effects of interleukin-1 and tumor necrosis factor on ketogenesis. Am. J. Physiol. Endocrinol. Metab..

[8] Dong H., Wang S., Jia Y., Ni Y., Zhang Y., Zhuang S., Shen X., Zhao R. (2013). Long-term effects of subacute ruminal acidosis (SARA) on milk quality and hepatic gene expression in lactating goats fed a high-concentrate diet. PLoS ONE.

[9] National Research Council (NRC) (2007). Nutrient Requirements of Small Ruminants: Sheep, Goats, Cervids, and New World Camelids.

[10] Hall M.B. (2009). Determination of starch, including maltooligosaccharides, in animal feeds: Comparison of methods and a method recommended for AOAC collaborative study. J. AOAC Int..

[11] Xu H., Bionaz M., Sloboda D.M., Ehrlich L., Li S., Newnham J.P., Dudenhausen J.W., Henrich W., Plagemann A., Challis J.R. (2015). The dilution effect and the importance of selecting the right internal control genes for RT-qPCR: A paradigmatic approach in fetal sheep. BMC Res. Notes.

[12] Janovick-Guretzky N.A., Dann H.M., Carlson D.B., Murphy M.R., Loor J.J., Drackley J.K. (2007). Housekeeping Gene Expression in Bovine Liver is Affected by Physiological State, Feed Intake, and Dietary Treatment. J. Dairy Sci..

[13] Chishti G.A., Salfer I.J., Suarez-Mena F.X., Harvatine K.J., Heinrichs A.J. (2019). Relationships between physical form of oats in starter, rumen pH, and volatile fatty acids on hepatic expression of genes involved in metabolism and inflammation in dairy calves. J. Dairy Sci..

[14] Xie F., Xiao P., Chen D., Xu L., Zhang B. (2012). miRDeepFinder: A miRNA analysis tool for deep sequencing of plant small RNAs. Plant Mol. Biol..

[15] Steele M.A., Greenwood S.L., Croom J., McBride B.W. (2012). An increase in dietary non-structural carbohydrates alters the structure and metabolism of the rumen epithelium in lambs. Can. J. Anim. Sci..

[16] Odongo N., AlZahal O., Lindinger M., Duffield T., Valdes E., Terrell S., McBride B. (2006). Effects of mild heat stress and grain challenge on acid-base balance and rumen tissue histology in lambs. J. Anim. Sci..

[17] Gebretsadkan T.K., Ambachew G., Birhaneselassie H. (2015). The comparison between microhematocrit and automated methods for hematocrit determination. Int. J. Blood Res. Disord.

[18] Naylor J.M., Kronfeld D.S. (1977). Refractometry as a measure of the immunoglobulin status of the newborn dairy calf: Comparison with the zinc sulfate turbidity test and single radial immunodiffusion. Am. J. Vet. Res..

[19] McCarthy M.M., Yasui T., Felippe M.J.B., Overton T.R. (2016). Associations between the degree of early lactation inflammation and performance, metabolism, and immune function in dairy cows. J. Dairy Sci..

[20] Osorio J.S., Trevisi E., Li C., Drackley J.K., Socha M.T., Loor J.J. (2016). Supplementing Zn, Mn, and Cu from amino acid complexes and Co from cobalt glucoheptonate during the peripartal period benefits postpartal cow performance and blood neutrophil function. J. Dairy Sci..

[21] Maxie G. (2015). Jubb, Kennedy & Palmer’s Pathology of Domestic Animals-E-Book (Volume 2).

[22] Hinders R.G., Owen F.G. (1965). Relation of ruminal parakeratosis development to volatile fatty acid absorption. J. Dairy Sci..

[23] Lorenz I. (2019). Ruminal Parakeratosis. Merck Veterinary Manual.

[24] Penner G.B., Taniguchi M., Guan L.L., Beauchemin K.A., Oba M. (2009). Effect of dietary forage to concentrate ratio on volatile fatty acid absorption and the expression of genes related to volatile fatty acid absorption and metabolism in ruminal tissue. J. Dairy Sci..

[25] Liu L., Sun D., Mao S., Zhu W., Liu J. (2019). Infusion of sodium butyrate promotes rumen papillae growth and enhances expression of genes related to rumen epithelial VFA uptake and metabolism in neonatal twin lambs. J. Anim Sci..

[26] Sander E.G., Warner R.G., Harrison H.N., Loosli J.K. (1959). The stimulatory effect of sodium butyrate and sodium propionate on the development of rumen Mmcosa in the young calf. J. Dairy Sci..

[27] Minuti A., Ahmed S., Trevisi E., Piccioli-Cappelli F., Bertoni G., Jahan N., Bani P. (2014). Experimental acute rumen acidosis in sheep: Consequences on clinical, rumen, and gastrointestinal permeability conditions and blood chemistry1. J. Anim Sci..

[28] Bevans D.W., Beauchemin K.A., Schwartzkopf-Genswein K.S., McKinnon J.J., McAllister T.A. (2005). Effect of rapid or gradual grain adaptation on subacute acidosis and feed intake by feedlot cattle. J. Anim Sci..

[29] Chang G., Zhang K., Xu T., Jin D., Seyfert H.-M., Shen X., Zhuang S. (2015). Feeding a high-grain diet reduces the percentage of LPS clearance and enhances immune gene expression in goat liver. BMC Vet. Res..

[30] Rossi E. (2005). Hepcidin—the iron regulatory hormone. Clin. Biochemist. Rev..

[31] Nemeth E., Ganz T. (2009). The role of hepcidin in iron metabolism. Acta Haematol..

[32] Nicolas G., Chauvet C., Viatte L., Danan J.L., Bigard X., Devaux I., Beaumont C., Kahn A., Vaulont S. (2002). The gene encoding the iron regulatory peptide hepcidin is regulated by anemia, hypoxia, and inflammation. J. Clin. Invest..

[33] da Silva C.B., Wolkmer P., Paim F.C., da Silva A.S., Siqueira L.C., de Souza C.L., França R.T., Dornelles G.L., Duarte M.M.M.F., Monteiro S.G. (2013). Iron metabolism and its relationship to anemia and immune system in Trypanosoma evansi infected rats. Exp. Parasitol..

[34] Zebeli Q., Dunn S.M., Ametaj B.N. (2011). Perturbations of plasma metabolites correlated with the rise of rumen endotoxin in dairy cows fed diets rich in easily degradable carbohydrates. J. Dairy Sci..

[35] Steele M.A., Vandervoort G., AlZahal O., Hook S.E., Matthews J.C., McBride B.W. (2011). Rumen epithelial adaptation to high-grain diets involves the coordinated regulation of genes involved in cholesterol homeostasis. Physiol. Genom..

[36] Rodríguez-Lecompte J.C., Kroeker A.D., Ceballos-Márquez A., Li S., Plaizier J.C., Gomez D.E. (2014). Evaluation of the systemic innate immune response and metabolic alterations of nonlactating cows with diet-induced subacute ruminal acidosis. J. Dairy Sci..

[37] Velez J., Donkin S. (2005). Feed restriction induces pyruvate carboxylase but not phosphoenolpyruvate carboxykinase in dairy cows. J. Dairy Sci..

[38] Overton T.R., Drackley J.K., Ottemann-Abbamonte C.J., Beaulieu A.D., Emmert L.S., Clark J.H. (1999). Substrate utilization for hepatic gluconeogenesis is altered by increased glucose demand in ruminants. J. Anim Sci..

[39] Aschenbach J.R., Kristensen N.B., Donkin S.S., Hammon H.M., Penner G.B. (2010). Gluconeogenesis in dairy cows: The secret of making sweet milk from sour dough. IUBMB Life.

[40] Penner G.B., Aschenbach J.R., Gäbel G., Rackwitz R., Oba M. (2009). Epithelial capacity for apical uptake of short chain fatty acids is a key determinant for intraruminal pH and the susceptibility to subacute ruminal acidosis in sheep. J. Nutr..

[41] Greenfield R., Cecava M., Donkin S. (2000). Changes in mRNA Expression for Gluconeogenic Enzymes in liver of dairy cattle during the transition to lactation 1. J. Dairy Sci..

