# Supplementary material for: Impacts of Time-Fed Concentrate-Based Diets on Plasma Metabolites, Rumen Histology, and mRNA Expression of Hepatic Enzymes of Wethers
Source: Animals (Basel). 2020 Apr 15;10(4):686. doi: 10.3390/ani10040686 (PMC7222829; doi:10.3390/ani10040686)
Supplement: Supplementary file 1 [file animals-10-00686-s001.pdf]

animals

Table S1. Box-Cox transformation received by mRNA expression of genes used in this study.

Gene	λ	Transformation
SAA	0	ln(x)
HPT	0	ln(x)
ALB	1	No [1]
HAMP	0.5	Sqrt(x)
HMGCR	−0.5	1/Sqrt(x)
HMGCS-2	0.5	Sqrt(x)
PC	−1	1/x

ln = natural logarithm, Sqrt = Square root, *SAA* = Serum α-amyloid, *HPT* = Haptoglobin, *ALB* = Albumin, *HAMP* = Hepcidin, *HMGCR* = 3-hydroxy-3-methylglutaryl-CoA reductase, *HMGCS-2* = 3-hydroxy-3-methyl-glutaryl CoA-synthase, *PC* = Pyruvate carboxylase. [1] No transformation was required.

Table S2. Least square means ± SEM of mRNA expression of Serum α-amyloid and Haptoglobin before back transformation.

**Gene**	**P1**	**P2**	**P3**
SAA	−3.28 ± 0.829	−0.96 ± 0.829	−2.34 ± 0.741
HPT	−0.80 ± 0.730	0.93 ± 0.730	−1.19 ± 0.653

SAA = Serum α-amyloid, HPT = Haptoglobin.
